# B Cell Response Induced by SARS-CoV-2 Infection Is Boosted by the BNT162b2 Vaccine in Primary Antibody Deficiencies

**DOI:** 10.3390/cells10112915

**Published:** 2021-10-27

**Authors:** Federica Pulvirenti, Ane Fernandez Salinas, Cinzia Milito, Sara Terreri, Eva Piano Mortari, Concetta Quintarelli, Stefano Di Cecca, Gianluca Lagnese, Alessandra Punziano, Marika Guercio, Livia Bonanni, Stefania Auria, Francesca Villani, Christian Albano, Franco Locatelli, Giuseppe Spadaro, Rita Carsetti, Isabella Quinti

**Affiliations:** 1 Regional Reference Centre for Primary Immune Deficiencies, Azienda Ospedaliera Universitaria Policlinico Umberto I, 00185 Rome, Italy; federica.pulvirenti.md@gmail.com (F.P.); livia.bonanni@inwind.it (L.B.); stefaniaauria@gmail.com (S.A.); francesca.villani@uniroma1.it (F.V.); 2 Department of Molecular Medicine, Sapienza University of Rome, 00185 Rome, Italy; anefdz@hotmail.com (A.F.S.); cinzia.milito@uniroma1.it (C.M.); 3 Diagnostic Immunology Research Unit, Multimodal Medicine Research Area, Bambino Gesù Children’s Hospital, IRCCS, Viale di San Paolo, 00146 Rome, Italy; sara.terreri87@gmail.com (S.T.); eva.pianomortari@gmail.com (E.P.M.); christian.albano@opbg.net (C.A.); rita.carsetti@opbg.net (R.C.); 4 Department Onco-Haematology, and Cell and Gene Therapy, Bambino Gesù Children Hospital, IRCCS, 00116 Rome, Italy; concetta.quintarelli@opbg.net (C.Q.); stefano.dicecca@opbg.net (S.D.C.); marika.guercio@opbg.net (M.G.); franco.locatelli@uniroma1.it (F.L.); 5 Department of Clinical Medicine and Surgery, University of Naples Federico II, 80131 Naples, Italy; 6 Department of Translational Medical Sciences, University of Naples Federico II, 80131 Naples, Italy; g.lagnese93@gmail.com (G.L.); a.punziano@studenti.unina.it (A.P.); spadaro@unina.it (G.S.); 7 Dipartimento Materno-Infantile e Scienze Urologiche, Sapienza University of Rome, 00185 Rome, Italy

**Keywords:** common variable immunodeficiencies, SARS-CoV-2, COVID-1, BNT162b2, vaccine, third dose, memory B cells, spike protein, antibody response

## Abstract

Background: Patients with primary antibody deficiencies are at risk in the current COVID-19 pandemic due to their impaired response to infection and vaccination. Specifically, patients with common variable immunodeficiency (CVID) generated poor spike-specific antibody and T cell responses after immunization. Methods: Thirty-four CVID convalescent patients after SARS-CoV-2 infection, 38 CVID patients immunized with two doses of the BNT162b2 vaccine, and 20 SARS-CoV-2 CVID convalescents later and immunized with BNT162b2 were analyzed for the anti-spike IgG production and the generation of spike-specific memory B cells and T cells. Results: Spike-specific IgG was induced more frequently after infection than after vaccination (82% vs. 34%). The antibody response was boosted in convalescents by vaccination. Although immunized patients generated atypical memory B cells possibly by extra-follicular or incomplete germinal center reactions, convalescents responded to infection by generating spike-specific memory B cells that were improved by the subsequent immunization. Poor spike-specific T cell responses were measured independently from the immunological challenge. Conclusions: SARS-CoV-2 infection primed a more efficient classical memory B cell response, whereas the BNT162b2 vaccine induced non-canonical B cell responses in CVID. Natural infection responses were boosted by subsequent immunization, suggesting the possibility to further stimulate the immune response by additional vaccine doses in CVID.

## 1. Introduction

Due to the severely impaired immune response to infection and immunization, patients with primary antibody defects (PADs) may be at increased risk for severe or prolonged infections [1,2]. In particular, patients with common variable immunodeficiencies (CVIDs), the most common symptomatic PAD, have an impaired response to infections and vaccination, severely reduced circulating class-switched memory B cells (MBCs), and strongly decreased plasmablast/plasma cell production, associated with impaired post-germinal center (GC) B cell maturation and differentiation in blood and secondary lymphoid tissues [3,4].

Since the start of the SARS-CoV-2 pandemic, clinical descriptions of COVID-19 in CVID patients are expanding, with a clinical presentation varying from asymptomatic or mild symptoms to death [5,6,7,8,9,10,11]. In Italy, we demonstrated that CVID patients have a cumulative incidence and an infection fatality rate similar to the SARS-CoV-2-positive general population [12]. Different from the general population, CVID patients display a lower median age at death and do not present the same risk factors predisposing to severe COVID-19 [13,14,15] with the exception of the underlying chronic lung disease (CLD) [16].

Immunization is the safest and most effective tool to achieve a protective response against SARS-CoV-2 infection and to terminate the pandemic [17,18]. In immunocompetent individuals, mRNA vaccine elicits high SARS-CoV-2-neutralizing antibodies and robust antigen-specific CD8+ and CD4+ T cell responses [19,20]. Clinical trials showed an effectiveness of almost 95% in preventing severe COVID-19 disease [17]. In Italy, COVID-19 immunization has been made available for fragile patients since March 2021 [21]. Thanks to its safety profile, SARS-CoV-2 immunization is highly recommended also in PAD patients [22]. However, due to the immune defect, their responses to vaccines are variable [23,24].

Here, we compared the adaptive responses induced by natural SARS-CoV-2 infection and immunization with an mRNA vaccine in patients with CVID. Our results showed that vaccination and infection prime different B cells responses and that the humoral immune response induced by natural infection can be significantly enhanced by subsequent immunization.

## 2. Methods

### 2.1. Study Design and Patients

Interventional study carried out in two groups of CVID patients: 34 subjects previously infected by SARS-CoV-2 (thereafter indicated as convalescent) and 38 subjects naive to SARS-CoV-2 infection, who were immunized by the BNT162b2 vaccine (reported as immunized). Participants were diagnosed as having CVID according to the ESID criteria [25]. Eligible patients were informed on the study, including its safety profile and supply procedures.

SARS-CoV-2-positive patients were identified by RT-PCR on nasopharyngeal swabs within 48 h from the symptom onset or in case of family contact. COVID-19 clinical symptoms, demographic characteristics, and comorbidities data were collected by study physicians.

In the immunized group, the BNT162b2 vaccine was administered in two doses, with 21 days apart. Blood samples were obtained for serological and cellular immunity assessment at baseline (BL) before immunization and seven days after the second dose. Samples from SARS-CoV-2-convalescent patients were obtained after a negative RT-PCR.

Blood samples were also collected in a group of 20/34 convalescent patients who underwent immunization with a single dose of BNT162b2 vaccine (indicated as convalescent/immunized). During the study, the participants were allowed to continue their therapies, including immunoglobulin substitution as a standard therapy for the underlying antibody deficiency. The study was approved by the Ethical Committee of the Sapienza University of Rome (Prot. 0521/2020, 13 July 2020) and was performed in accordance with the Good Clinical Practice guidelines, the International Conference on Harmonization guidelines, and the most recent version of the Declaration of Helsinki.

### 2.2. ELISA for Specific IgG Detection

A semi-quantitative in vitro determination of human IgG antibodies against the SARS-CoV-2 (S1) was performed on serum samples by using the anti-SARS-CoV-2 spike ELISA (EUROIMMUN, Lübeck, Germany), according to the manufacturer’s instructions. Values were then normalized by comparison with a calibrator. Results were obtained by calculating the ratio between the extinction of samples and the extinction of the calibrator. Results are reported as the ratio between the optical density (OD) from the sample and the OD from the calibrator. The ratio interpretation was as follows: negative, <0.8; borderline, ≥0.8 to <1.1; positive, ≥1.1.

### 2.3. Detection of SARS-CoV-2-Specific B Cells 

The analysis of spike-specific cellular immunity was performed in 23, 17, and 3 selected immunized, convalescent, and convalescent/immunized patients, respectively. We did not analyze spike-specific B cells frequency in 15 immunized, 17 convalescents, and 17 convalescent/immunized patients, due to the lack of blood samples. Peripheral blood mononuclear cells (PBMCs) were isolated by Ficoll Paque™ Plus 206 (Amersham PharmaciaBiotech, Amersham, United Kingdom) density-gradient centrifugation and immediately frozen and stored in liquid nitrogen until use. The freezing medium contained 90% fetal bovine serum (FBS, Gibco, MA, USA) and 10% DMSO (Sigma-Aldrich, MI, USA). To detect SARS-CoV-2-specific B cells, biotinylated protein antigens were individually multimerized with fluorescently labelled streptavidin at 4 °C for 1 h, as previously described [26]. Briefly, a recombinant biotinylated SARS-CoV-2 spike (S1 + S2; aa16-1211) was purchased from R&D systems (Minnesota, USA) (BT10549).

B cell subsets were identified based on the expression of CD19, CD27, CD24, and CD38 markers by flow cytometry performed before freezing cell samples. MBCs were defined as CD19+CD24+CD27+CD38– and atypical MBCs (ATMs) were identified as CD19+CD27–CD24–CD38– [27,28]. Stained PBMC samples were acquired on FACs LSRFortessa (BD Bioscience, NJ, USA). At least 4 × 10^6^ cells were acquired and analyzed using FlowJo10.7.1 (BD Bioscience). The phenotype analysis of antigen-specific B cells was only performed in subjects with at least 10 cells detected in the corresponding antigen-specific gate. Figure S1 shows the gating strategy.

### 2.4. Detection of SARS-CoV-2-Specific T Cells

T cell subsets were identified based on the expression of CD3, CD4, CD8, and CD45 markers by flow cytometry performed before freezing cell samples. The analysis of spike-specific cellular immunity was performed in 9 immunized, 15 convalescent, and 3 selected convalescent/immunized patients. We did not analyze spike-specific T cells frequency in 19 immunized, 19 convalescents, and 17 convalescent/immunized patients, due to the lack of blood samples.

We used an IFNγ ELISpot assay (Mabtech, Nacka Strand, Sweden), as previously described [29]. Briefly, T cells were plated in duplicate, with 2 × 10^5^ cells/well, stimulated with 1 µg/mL CRUDE PepMix™ SARS-CoV-2 (Spike Glycoprotein, JPT Peptide Technologies, Berlin, Germany) and incubated at 37 °C for 24 h. In all experiments, T cells were also incubated with serum-free CellGenix^TM^ GMP (Cell Genix, GMBH, Freiburg, Germany) as a negative control. As a positive control, PBMCs were stimulated with 5 µg/mL of phytohemoagglutinin-P (PHA, Sigma-Aldrich). The *IFNγ*+ spot-forming unit (SFU) was counted with EliScan (Epson) by Automated ELisa-Spot Assay Video Analysis Systems (A.EL.VIS GmbH, Hannover, Germany). Data were presented as the percentage of IFNγ SFUs obtained after pepMix stimulation, compared to that of the total SFUs obtained in the positive control condition (PHA).

### 2.5. Statistical Analysis

Data obtained post-immunization, post-SARS-CoV-2 infection, and post-immunization in convalescent participants were separately analyzed. Only for the spike-specific IgG evaluation, data from patients treated with SARS-CoV-2 monoclonal antibodies (MoAbs) were separately analyzed. Demographics were summarized with descriptive statistics (continuous values of the median and the interquartile range IQR). A univariate analysis assessed the impact of variables of interest. Values were compared by two-tailed Mann–Whitney U-test or by Wilcoxon matched-pairs signed-rank test (convalescents vs. convalescents/immunized). Data were represented as individual data and median data (horizontal lines). The dotted area represented the IQR range recorded in healthy donors, as previously reported [26]. The comparison between continuous parameters was assessed by simple linear regression analysis. Differences were deemed significant when *p* < 0.05. The Statistical Package for Social Sciences version 15 (SPSS Inc., 233 South Wacker Drive, 11th Floor, Chicago, USA) was used for the analysis.

## 3. Results

### 3.1. Patients 

Blood samples were collected from 34 SARS-CoV-2 convalescent CVID patients (median age = 49.5 years; IQR = 45–59; 47% females) after a median of 86.5 days (IQR = 51.7–120) days from the first positive RT-PCR by nasopharyngeal swab. Convalescent patients were compared to 38 CVID patients naïve to SARS-CoV-2 who were immunized by two doses of the BNT162b2 vaccine (median age = 54 years; IQR = 42–60; 66% females). Demographic and immunological characteristics and comorbidities in the study groups are summarized in Table 1.

The clinical symptoms of COVID-19, treatments, and outcomes in the convalescents are summarized in Table 2. According to the World Health Organization criteria 2020, 14 (41%) patients were classified as asymptomatic COVID-19, 9 (26%) were classified as mild, 9 (26%) were classified as moderate, and 2 (7%) were classified as severe COVID-19 [30]. The median time of SARS-CoV-2 RT-PCR positivity was 27 days (IQR = 17–51). Nine patients developed pneumonia, and no patients were admitted to the ICU. Fourteen out of 34 patients received COVID-19 treatment. Five of them were treated with SARS-CoV-2 MoAbs (median age = 59 years; IQR = 50–64; 40% females). Infection outcome was favorable in all patients, with the exception of two patients who required long-term oxygen therapy due to the worsening in their underlying CLD.

Among convalescents, 20 patients were immunized by BNT162b2 COVID-19 vaccine after a median of 94.2 days (IQR = 80.2–140.5) from the first SARS-CoV-2-negative test. In these convalescent/immunized patients, blood samples were collected after a median of 21 days (IQR = 21–45) from immunization. The immunized patients, convalescent patients, and convalescent/immunized patients did not differ for demographics and PAD comorbidities. Only CLD was more frequently observed in the SARS-CoV-2-infected patients (Table 1).

### 3.2. SARS-CoV-2 Antibodies

**Figure 1:**
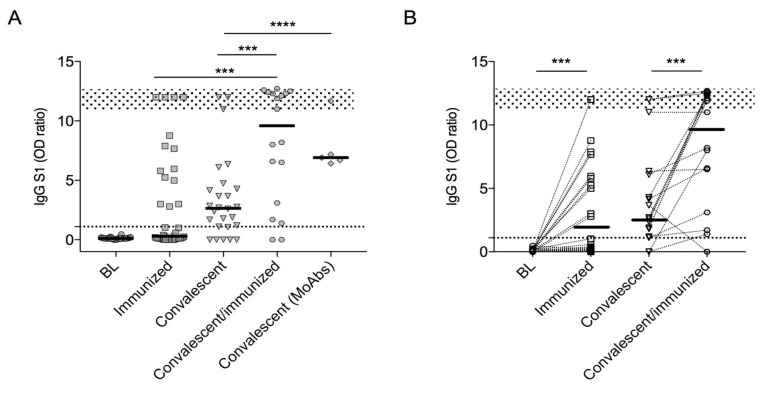
Spike-specific IgG levels in the CVID cohort (panel (**A**)) Data are shown at baseline (BL) after immunization (immunized, *n* = 38), after infection (convalescent, *n* = 34), and after immunization in convalescents (convalescent/immunized *n* = 20). Data from monoclonal antibodies (MoAbs, *n* = 5) recipients are shown separately. Changes in individual data points of spike-specific IgG antibodies from BL to post-immunization and from SARS-CoV-2 recovery to post-immunization are shown in panel (**B**). The positive cut-off values are represented by a dashed line. For each group, medians are plotted as horizontal bars. The dotted area represents the IQR range in healthy donors (HD) after immunization as previously reported [26]. **** *p* < 0.0001; *** *p* < 0.001; *p*-value represents the level of significance by two-tailed Mann–Whitney U-test (immunized vs convalescent/immunized and convalescent vs convalescent (MoAbs)) or by Wilcoxon matched-pairs signed-rank test (BL vs immunized and convalescents vs convalescents/immunized). Abbreviation: BL baseline, IgG S1: Spike specific IgG, OD: optical density, MoAbs: Monoclonal antibodies treatment.

Anti-spike IgG response was assessed in immunized, convalescent, and convalescents/immunization. In the three groups, the values of IgG S1 antibodies were significantly increased in comparison to the BL values. After infection, a higher number of convalescent CVID patients showed a detectable anti-spike IgG antibody response (IgG S1 > 1.1 OD ratio; 24/29, 82%) in comparison to the number of responders in the immunized group (14/38; 34%; *p* = 0.0002). However, the median anti-spike IgG serum level was not significantly higher in the convalescent group than in the immunized group (*p* = 0.553; Figure 1A).

The highest IgG S1 response was observed in the group of convalescents/immunized, where the post-infection antibody response was boosted after immunization (*p* = 0.001), and in infected patients who underwent anti-spike MoAbs treatment. To note, two out of five convalescent patients who were seronegative after SARS-CoV-2 infection seroconverted after immunization. The summary of IgG S1 in CVID was reported in Table 3, showing spike-specific IgG antibodies in 38 CVID patients before (BL) and one week after the second dose of the BNT162b2 vaccine (immunized) and in 20 CVID patients after recovery from SARS-CoV2 infection (convalescent) and after one dose of the BNT162b2 vaccine (convalescent/immunized).

The individual data points of spike-specific IgG antibodies from BL to post-immunization and from SARS-CoV-2 recovery to post-immunization after one dose of the BNT162b2 vaccine (convalescent/immunized) are presented in Figure 1, panel B. The positive cut-off values are represented by a dashed line.

In our cohort, patients who did not mount a detectable antibody response after immunization had a lower frequency of switched MBCs (*p* = 0.0005) and lower serum IgA and IgM levels (*p* = 0.0002 and *p* = 0.042, respectively). In detail, 10% of those with low frequencies of switched MBCs (<2%) showed a detectable humoral response. Differently, in the convalescents, we could not identify any immunological or clinical signatures associated with the different antibody response after infection. To note, 50% of patients with low frequencies of switched MBCs were able to mount a detectable humoral response. The convalescents with more severe COVID-19 courses did not develop more frequently a detectable IgG S1 response (Table S1).

### 3.3. Spike-Specific SARS-CoV-2 MBCs

High specificity and affinity are the most important characteristics of protective MBCs, generated by the adaptive immune system in response to infection or vaccination in the GC [31], thanks to the mechanisms of somatic mutation and affinity selection [32]. After immunization or infection, ATMs become transiently detectable in the peripheral blood, mostly generated by extrafollicular reactions where antigen selection does not occur [33].

Recently, we showed that vaccination induces spike-specific (S+) ATMs in about one-third of patients in CVID [26], different from healthy subjects who respond by generating S+ MBCs (Figure S2) [34].

**Figure 2:**
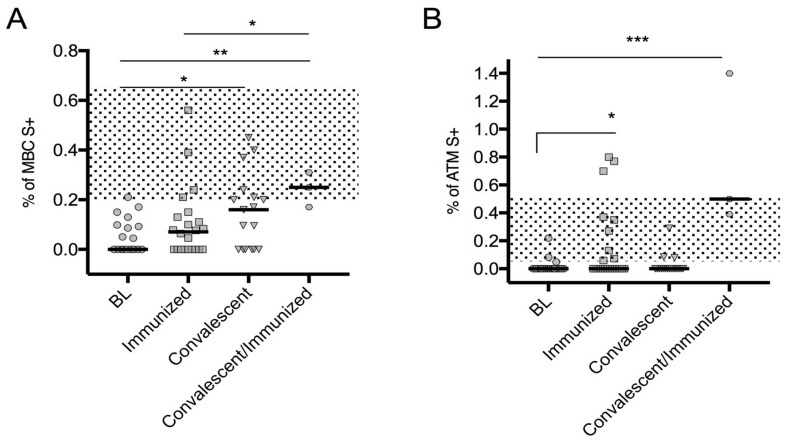
Spike-specific MBC subsets in the CVID cohort. Spike-specific MBCs (**A**) and ATMs (**B**) data are shown at BL *(n* = 23), after immunization (immunized, *n* = 23), after SARS-CoV-2 infection (convalescent, *n* = 17), and after immunization in SARS-CoV-2 convalescents (convalescent/immunized, *n* = 3). Medians are plotted as horizontal bars. The dotted area represents the IQR range in HD after immunization as previously reported [26]. Levels of significance were measured by two-tailed Mann–Whitney U-test and for Wilcoxon matched-pairs signed-rank test (comparison between convalescents and convalescents/immunized only): *** *p* < 0.001; ** *p* < 0.01; * *p* < 0.05. Abbreviations: BL, baseline; MBC S+, spike-specific memory B cells; ATM S+, atypical memory B cells.

Here, we recorded that convalescent CVID generated S+ MBCs cells, but not S+ ATMs. Remarkably, after immunization, the convalescents generated also S+ ATMs and developed an even more evident classical spike-specific MBC response. In this cohort, we confirmed that immunized CVID patients generated specific S+ ATMs, but not S+ MBCs (Figure 2).

The spike-specific B cells subset frequencies were similar among patients with severe/moderate COVID-19 or mild/asymptomatic infection (data not shown). Moreover, the B cells subset frequencies were similar in patients when grouped accordingly to their ability to develop a detectable IgG S1 antibody response, both after immunization and after infection (Table S1). No linear correlation was observed between the frequencies of the specific B cells subset and IgG S1 levels (R = 0.003; *p* = 0.855). The Frequencies of MBCs++ and ATMs S++ were not shown, because CVID patients are unable to produce them.

### 3.4. SARS-CoV-2 T Cell-Specific ELISpot Response

**Figure 3:**
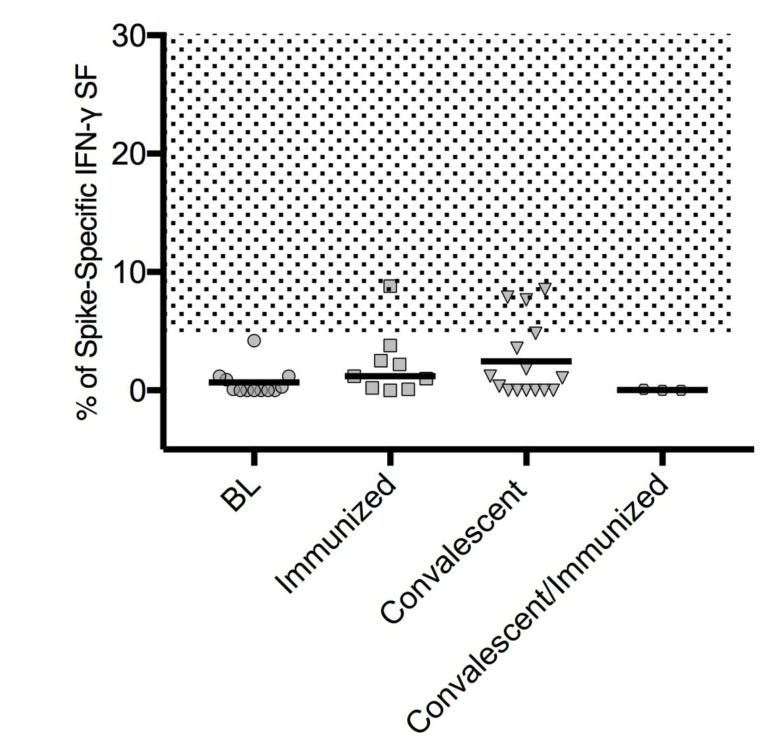
SARS-CoV-2 T cell-specific response in CVID patients. Data are shown as the percentage of increment *IFNγ* production after T cells stimulation with the spike SARS-CoV-2 protein at BL (*n* = 9), after immunization (immunized, *n* = 9), after COVID-19 recovery (convalescent, *n* = 15), and in convalescents after immunization (convalescent/immunized, *n* = 3). Percentage distributions and medians (horizontal lines) are shown. The dotted area represents the IQR range in HD after immunization as previously reported [26]. Values were compared by two-tailed Mann–Whitney U-test or Wilcoxon matched-pairs signed-rank test (comparison between convalescents and convalescents/immunized only). Abbreviations: BL, baseline; IFN-*γ*, interferon gamma spot-forming unit.

T cell activity was indirectly analyzed by measuring the concentration of *IFNγ* secreted by activated lymphocytes after 24 h of the in vitro stimulation. On the basis of the cut-off of spot increments of *IFNγ,* we calculated the positive SARS-CoV-2 T cell-specific response after stimulation with the spike SARS-CoV-2. The median percentage of the positive response was 1.2% in immunized CVID (IQR = 0.15–3.15) and 1.04% (IQR = 0.0–4.8) in convalescents. These values were lower than previously reported in the healthy donors (median = 9%; IQR = 5–30) [25]. The absence of specific T cell responses was also observed in convalescent/immunized CVID patients (Figure 3).

## 4. Discussion

In CVID, the impaired immune response after infections and immunization accounts for the high infection susceptibility [3], requiring specific prevention and treatment strategies to minimize morbidity and mortality. In the current SARS-CoV-2 pandemic, 6.3% of CVID patients attending our collaborative centers became infected. Consistent with other reports [10,12,35], about 65% of patients had a mild or asymptomatic COVID-19 course. Since vaccination became available, almost all 335 CVID patients from this cohort were immunized with an mRNA COVID-19 vaccine (BNT162b2). A single dose of mRNA vaccine was administered in 20 convalescent patients starting from 30 days from the infection recovery.

This study was planned in order to evaluate if natural SARS-CoV-2 infection and immunization induced different adaptive immune responses in CVID patients. Our results showed that vaccination and infection primed different B cells responses. Moreover, the humoral immune response induced by natural infection was significantly enhanced by subsequent immunization. The antibody response was influenced neither by the severity of COVID-19 nor by the duration of viral replication.

Natural infection generated a detectable antibody IgG response in more than 80% of convalescent patients, a surprisingly higher proportion in comparison to in fully immunized CVID patients, who seroconverted only in 34% of cases. Here, we confirmed our previous data showing low adaptive responses to immunization with the BNT162b2 vaccine in CVID subjects [26]. Our data contrasted with the high frequency of response reported in small heterogeneous cohorts of immunized PAD patients [35,36,37,38].

However, the improved antibody response by a single vaccine dose in convalescent patients is in line with observations in convalescent/vaccinated immunocompetent subjects [39,40]. In our cohort, immunization was able to induce a detectable antibody response also in two out of five CVID seronegative convalescents. An analogous increase of the seroconversion rate after immunization has been recently observed also in other SARS-CoV-2 convalescent fragile patients, including organ transplant recipients [20,40].

The differences in antibody response between immunization and natural infection mirrored the B cell subsets phenotype and the spike-specific B cell responses. The frequency of switched CD27+ MBCs affected the antibody response in vaccinated, but not in convalescent CVID, patients, pointing out that natural infection can induce an antibody response also in those having a severe B memory cell defect. More importantly, only convalescents generated spike-specific MBCs, whereas immunized generated spike-specific ATM only, revealing alternative patterns of the B cell response. Interestingly, we recorded that spike-specific ATMs were also elicited by immunization in convalescents.

Impaired post-GC B cell maturation, severely reduced circulating class-switched MBCs, and persistent circulating ATMs are the most consistent defects in CVID [4,40,41]. In comparison to classical MBCs, ATMs display hypo-responsiveness to B cell receptor (BCR) stimulation, upregulation of inhibitory receptors, and limited antibody secretion upon stimulation [42,43].

Different from the spike-specific B cell response observed in immunized CVID, the B cell response in convalescents CVID was consistent with that observed in immunized immunocompetent individuals, who generated spike-specific MBCs switching from the IgM+ to IgM– isotype, due to the process of affinity maturation and class-switching in the GC [26]. In immunocompetent subjects, it has been suggested that the infection generates more robust GC responses than the mRNA vaccine [44] and spike-specific B cell responses differ in immunized and convalescents with striking differences in the cell composition and transcriptional profiles of circulating immune cells, possibly linked to the different *milieu* induced by activated innate immune cells in infected patients [45]. Likewise, it is then possible to hypothesize a different pattern of response observed in immunized, convalescent, and convalescent/immunized patients, which is even more complex by the multiple innate [46,47,48] and adaptive immune abnormalities described in CVID [4,20,40,49,50,51,52]. Thus, also in CVID, the comparison of immune responses generated by the vaccine and the infection shed light on the difference between an antigen-driven response and an infection-driven response where the inflammation directs the subsequent adaptive immune response.

Different from what was observed in immunocompetent individuals after immunization and in small cohorts of PAD patients that mounted a robust antigen-specific CD8+ and CD4+ T cell responses after vaccination and natural infection [53,54], we recorded a poor T cell response after immunization, after infection, and after vaccination in convalescent patients. While in CVID influenza virus immunization generates specific T cells after multiple exposures to viral antigen, the poor spike-specific T cell response might be a consequence of a limited antigenic stimulation by a new pathogen, which has never been encountered before [55]. At present, it might be assumed that patients with poor T cell responses might require multiple doses or combinations of SARS-CoV-2 vaccines to obtain a possible protection. The main limitation of this study is the limited number of samples analyzed for specific B and T cell responses in convalescents who have been immunized. Further analysis is ongoing to assess in the future for the dynamic of the immune responses in this population of patients. Our observations underline the need of vaccination in convalescent CVID patients. Then, immunization by a single dose of mRNA vaccine should be recommended in all CVID convalescent patients from SARS-CoV-2 infection starting from 30 days from infection recovery [56]. For patients naive to infection, a third “booster” dose could be necessary to achieve a better immune response against COVID-19.

## Figures and Tables

**Table 1:** Demographics, immunological, and primary antibody defect (PAD)-related characteristics in common variable immunodeficiency (CVID) participants.

	Group 1 Immunized *n* = 38	Group 2 Convalescents *n* = 34	Group 3 Convalescent/Immunized *n* = 20	Groups 1 vs. 2 *p*-Value	Groups 1 vs. 3 *p*-Value	Groups 2 vs. 3 *p*-Value
Sex, *n* (%)	25 (66)	16 (47)	12 (60)	0.157	0.776	0.408
Age, years, median (IQR)	54.5 (42.5–60.0)	49.5 (44.7–59.2)	49.5 (44.2–62.7)	0.840	0.973	0.943
PAD-related complication						
Autoimmunity, *n* (%)	18 (47)	9 (26)	7 (35)	0.094	0.414	0.550
Chronic lung disease, *n* (%)	8 (21)	20 (58)	10 (50)	0.001	0.036	0.560
Cancer, *n* (%)	2 (5)	2 (6)	2 (10)	1.000	0.602	0.622
Immunosuppressive treatment, *n* (%)	10 (26)	3 (9)	2 (10)	0.069	0.186	1.000
IgG, g/L, median (IQR)	6.9 (5.8–8.1)	7.5 (6.5–8.8)	7.6 (7.2–8.8)	0.069	0.122	0.881
IgA, mg/dL, median (IQR)	7.0 (0–25.0)	6.0 (0–28.0)	4.5 (0–12.8)	0.541	0.599	0.401
IgM, mg/dL, median (IQR)	19.6 (4–42)	20.0 (5–25.1)	9.5 (4–25)	0.576	0.355	0.648
CD3+, cell/mm^3^, median (IQR)	924 (748–1512)	974 (675–1548)	879 (639–1412)	0.513	0.255	0.335
CD4+, cell/mm^3^, median (IQR)	452 (356–811)	511 (356–811)	479 (335–838)	0.756	0.801	0.992
CD8+, cell/mm^3^, median (IQR)	421 (186–692)	369 (269–710)	297 (242–521)	0.741	0.281	0.095
CD19+, cell/mm^3^, median (IQR)	87 (20–184)	91 (29–172)	55 (30–131)	0.564	0.150	0.272
CD19+CD27+ IgM–IgD–, %, median (IQR)	3.5 (1–7.7)	2.0 (0–5.0)	2.5 (0–5.5)	0.649	0.921	0.771
CD19+CD27+IgM–IgD–, cell/mm^3^, median (IQR)	1 (0–5.8)	1.6 (0–4.1)	2.3 (0.1–4.6)	0.480	0.474	0.739
Atypical MBC (ATM) CD19+CD24-CD27–CD38–CD21–, % of CD19+, median (IQR)	3.69 (2.8–8.1)	3.4 (2.3–5.2)	3.1 (2.9–4.1)	0.469	0.613	0.863

**Table 2:** SARS-CoV-2 infections, treatments, and outcomes in 34 CVID patients.

ID	Age (Range)	Time Range of SARS-CoV-2 Infection	SARS-CoV-2 Infection Severity	SARS-CoV-2Associated Symptoms	Pneumonia	Days of SARS-CoV-2 qPCR Positivity	Additional COVID-19 Specific Therapy	Outcome	BNT162b2 Immunization
1	30–39	March–May 2020	moderate	fever and cough dyspnea	yes	45	lopinavir/ritonavir, tocilizumab, and dexamethasone	recovery	no
2	50–59	June–September 2020	asymptomatic		no	79	no	recovery	yes, 180 days from infection
3	40–49	January–April 2021	asymptomatic		no	30	no	recovery	no
4	60–60	October–December 2020	asymptomatic		no	14	no	recovery	no
5	50–59	October–December 2020	asymptomatic		no	81	no	recovery	no
6	50–59	June–September 2020	asymptomatic		no	51	no	recovery	no
7	30–39	January–April 2021	moderate	fever, cough, and dyspnea	no	23	no	recovery	no
8	40–49	October–December 2020	moderate	fever, cough, and dyspnea	yes	75	lopinavir/ritonavir, tocilizumab, and dexamethasone	dyspnoea and O_2_ therapy	no
9	30–39	January–April 2021	mild	fever	no	21	dexamethasone	recovery	no
10	40–49	January–April 2021	moderate	fever, cough, and dyspnea	yes	22	remdesivir and dexamethasone	recovery	no
11	60–69	January–April 2021	mild	fever	no	17	bamlanivimab/ etesevimab	recovery	no
12	50–59	January–April 2021	moderate	fever, cough, and dyspnea	yes	112	calsirimab/imdevimab, remdesivir, and dexamethasone	recovery	no
13	40–49	January–April 2021	moderate	fever, cough, and dyspnea	yes	40	calsirimab/imdevimab, remdesivir, and dexamethasone	dyspnoea and O_2_ therapy	no
14	60–69	January–April 2021	asymptomatic		no	33	bamlanivimab/ etesevimab	recovery	no
15	50–59	January–April 2021	asymptomatic		no	23	bamlanivimab	recovery	no
16	40–49	October–December 2020	asymptomatic		no	NA	no	recovery	yes, 150 days from infection
17	30–39	October–December 2020	asymptomatic		no	NA	no	recovery	yes, 150 days from infection
18	40–49	January–April 2021	moderate	fever and mild dyspnea	yes	49	dexamethasone and azithromycin	recovery	yes, 120 days from infection
19	20–29	October–December 2020	asymptomatic		no	15	no	recovery	yes, 120 days from infection
20	50–59	March–May 2020	severe	fever, cough, and severe dyspnea	yes	10	lopinavir/ritonavir, azithromycin, and hydroxychloroquine	recovery	yes, 330 days from infection
21	60–69	October–December 2020	moderate	fever and mild dyspnea	yes	60	NA	recovery	yes, 150 days from infection
22	50–59	January–April 2021	mild	ageusia	no	9	no	recovery	yes, 90 days from infection
23	50–59	October–December 2020	mild	fever	no	NA	no	recovery	yes, 180 days from infection
24	40–49	January–April 2021	asymptomatic		no	22	no	recovery	yes, 90 days from infection
25	40–49	January–April 2021	asymptomatic		no	9	no	recovery	yes, 120 days from infection
26	50–59	October–December 2020	asymptomatic		no	16	no	recovery	yes, 120 days from infection
27	40–49	October–December 2020	mild	fever and cough	no	23	dexamethasone and azithromycin	recovery	yes, 150 days from infection
28	60–69	October–December 2020	mild	fever and arthralgia	no	20	no	recovery	yes, 150 days from infection
29	≥70	October–December 2020	moderate	fever, mild dyspnea, and ageusia	no	27	dexamethasone and azithromycin	dyspnoea	yes, 90 days from infection
30	40–49	October–December 2020	asymptomatic		no	30	no	recovery	yes, 150 days from infection
31	20–29	January–April 2021	mild	ageusia and anosmia	no	59	no	recovery	yes, 120 days from infection
32	60–69	October–December 2020	mild	anosmia	no	34	no	recovery	yes, 150 days from infection
33	60–69	January–April 2021	mild	arthralgia	no	12	no	dyspnoea	yes, 120 days from infection
34	40–49	October–December 2020	severe	fever and moderate dyspnea	yes	53	dexamethasone and azithromycin	recovery	yes, 120 days from infection

**Table 3:** SARS-CoV-2 anti-spike antibody immunoassay results and S1 memory B cells (MBCs) in CVID patients.

	IgG S1 (OD Ratio)	MBC S + (% of CD24 + CD27 + CD38–Inside CD19 + Cells)	ATM S + (% of CD24–CD27–CD38–CD21–Inside CD19+ Cells)
Baseline, median (IQR)	0.11 (0.08–0.18)	0 (0–0.98)	0 (0–0)
Immunized, median (IQR)	0.30 (0.09–5.39)	0.07 (0–0.13)	0 (0–0.27)
Convalescent, median (IQR)	2.5 (1.07–4.04)	0.16 (0.22)	0 (0–0)
Convalescent/immunized median (IQR)	9.6 (2.75–12.33)	0.25 (1.17–0.31)	0.50 (0.39–1.40)
MoAbs treatment, median (IQR)	6.91 (6.56–9.42)	Nap	Nap

Abbreviations: MBC S+, spike-specific memory B cells; ATM S+, spike-specific atypical memory B cells; Nap, not applicable.

## Data Availability

The data presented in this study are available on request from the corresponding author.

## Supplementary Materials

The following are available online at https://www.mdpi.com/article/10.3390/cells10112915/s1, Figure S1. Gating strategy to identify S+ and S++ MBCs and ATMs. Flow cytometry plots showing the staining patterns of SARS-CoV-2 antigen probes in MBCs (identified as CD24+CD27+CD38–) and ATMs (identified as CD24CD27–CD38–CD21–) populations in one HD and one CVID subjects. The color code identifies spike-positive MBCs (blue) and ATMs (pink). Figure S2. Spike-specific memory B cell subsets in the healthy cohort. Spike-specific MBCs (A) and ATMs (B) data are shown at BL (*n* = 28), after immunization (immunized, *n* = 28), and after immunization in SARS-CoV-2 convalescents (convalescent/immunized, *n* = 9). Medians are plotted as horizontal bars. Levels of significance by Wilcoxon matched-pairs signed-rank test or two-tailed Mann–Whitney U-test: **** *p* < 0.0001, *** *p* < 0.001, ** *p* < 0.01. Table S1. Demographic, clinical, and immunological characteristics of CVID enrolled, stratified by antibody response to a two-dose mRNA vaccine or to natural infection.
