# Supplementary material for: B Cell Response Induced by SARS-CoV-2 Infection Is Boosted by the BNT162b2 Vaccine in Primary Antibody Deficiencies
Source: Cells. 2021 Oct 27;10(11):2915. doi: 10.3390/cells10112915 (PMC8616496; doi:10.3390/cells10112915)
Supplement: Supplementary file 1 [file cells-10-02915-s001.zip › cells-1404156-supplementary.pdf]

Supplemental information. Figure S1. Gating strategy to identify S+ and S++ MBCs and ATMs. Flow cytometry plots showing the staining patterns of SARS-CoV-2 antigen probes in MBCs (identified as CD24+CD27+CD38-) and ATMs (identified as CD24-CD27-CD38-CD21-) populations in one HD and one CVID subjects. S+ are B cells Spike-PE+, but Spike-BUV395-; S++ are instead Spike-PE+ and Spike-BUV395+. The color code identifies Spike+ and Spike++ in MBCs (blue) and ATMs (pink).

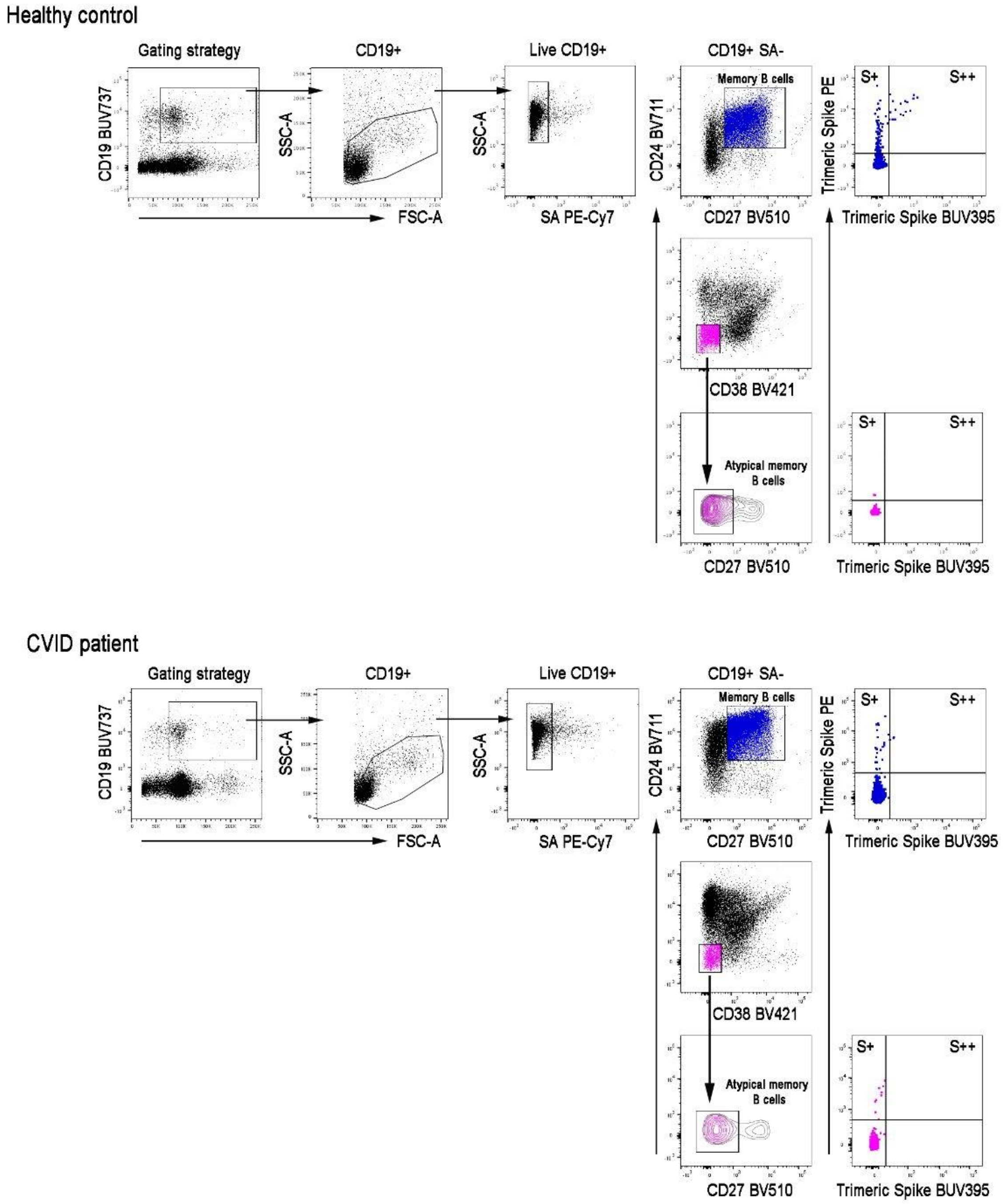

Figure S2. Spike-specific memory B cell subsets in the healthy cohort. Spike-specific MBCs (**A**) and ATMs (**B**) data are shown at BL *(n=28)*, after immunization (immunized, *n=28*), and after immunization in SARS-CoV-2 convalescents (convalescent/immunized, *n=9*). Medians are plotted as horizontal bars. Levels of significance by Wilcoxon matched-pairs signed-rank test or two-tailed Mann–Whitney U-test: **** p<0.0001, *** p<0.001, ** p<0.01.

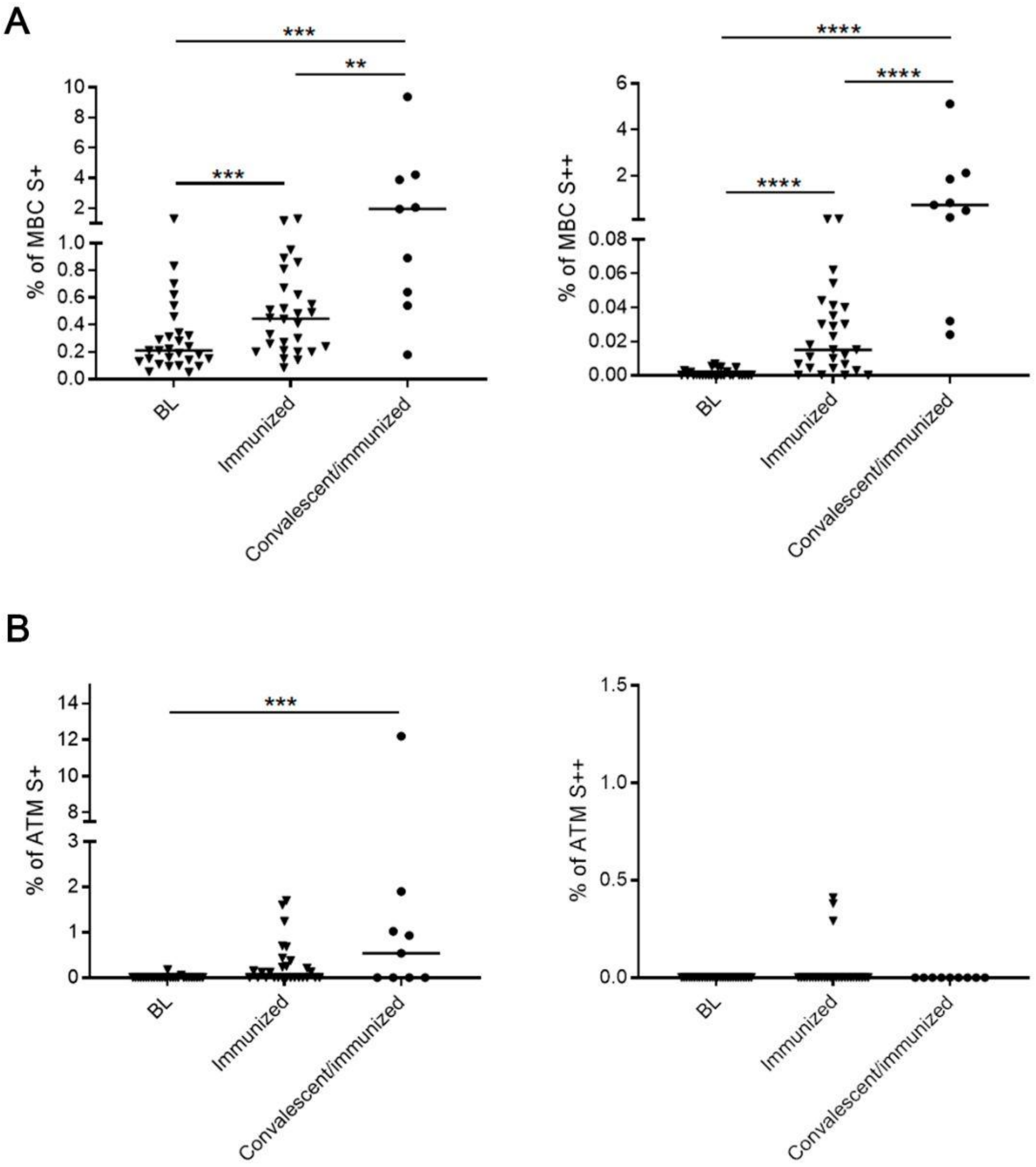

Table S1. Demographic, clinical and immunological characteristics of CVID enrolled, stratified by antibody response to a two-dose of mRNA vaccine or to natural infection. Responders are defined as having IgG S1 antibodies serum levels >1.1 OD ratio.

	**Immunized CVID**			**Convalescence CVID**		
	**Non responders n=24**	**Responders n=14**	**p value**	**Non responders n=5**	**Responders n=24**	**p value**
**Demographics**						
SEX, n (%)	17 (70%)	8 (57%)	0,486	3 (60)	11 (48)	0,651
Age, median (IQR)	52 (45,5-59,2)	54,5 (37,5-61,2)	0,944	55 (49-56)	48 (41-54)	0,451
**PAD-related conditions**						
Autoimmunity, n (%)	13 (54%)	5 (36%)	0,328	3 (60)	7 (29)	0,306
CLD, n (%)	5 (21%)	3 (21%)	1,000	2 (40)	13 (57)	0,651
Cancer, n (%)	2 (8%)	0 (0%)	0,522	1 (20)	1 (5)	0,342
Immuno-suppressive treatment, n (%)	7 (29%)	3 (21%)	0,715	0(0)	3 (14)	1,000
**Serum Ig level and immunophenotype**						
IgA (mg/dl), median (IQR)	4 (0-10)	25 (16-36)	0,0002	6 (0-42)	7 (0-42)	0,261
IgM (mg/dl), median (IQR)	13 (0-31,6)	36,6 (18-52)	0,042	24 (8,5-47,75)	25 (20,25-47,75)	0,283
CD3+ (cell/mm3), median (IQR)	924 (756,5-1387,5)	1029,5(844,70-1298,25)	0,780	917,5 (630-1447,2)	938 (765-1447,20)	0,455
CD4+ (cell/mm3), median (IQR)	452 (385,9-671,95)	490 (418,72-653,5)	0,362	509,60 (330-838,50)	540,20 (400,75 - 838,50)	0,455
CD8+ (cell/mm3), median (IQR)	418(195-741)	468,4 (341,42-592,5)	0,470	346 (243,60-533,40)	322,50 (279,90 - 533,40)	0,729
CD19+ (cell/mm3), median (IQR)	87,2(21-186,15)	81,8 (19,72-175,75)	0,877	76,30 (33,60-131)	91,50 (31,20-131)	0,266
CD27-IgM+IgD+ (%CD19+), median (IQR)	54(47-76)	55 (54-60)	0,978	27,5 (0,30-47,75)	36,50 (13,25-47,75)	0,180
CD27-IgM+IgD+ (cell/mm3), median (IQR)	51,8 (13-97,056)	43,3 (16,3-89,2)	0,492	10,21 (0,48 - 33,01)	13,76 (8,69-33,01)	0,245
CD27+IgM-IgD- (%CD19+), median (IQR)	1,1 (0,25-5)	15 (7,5-15,7)	0,0005	2,5 (0-5,50)	3 (0,65 - 5,50)	0,436
CD27+IgM-IgD-(cell/mm3), median (IQR)	1 (0-2)	9,0 (1,9-17,5)	0,025	2,32 (0-5,39)	3,10 (0,67-5,39)	0,184
**Specific immune response to Spike protein**						
IgG S+, OD ratio, median (IQR)	0,20 (0,0-0,26)	5,5 (3,5-7,2)	<0,0001	2,65 (0-4,74)	2,85 (1,8 - 4,7)	0,011
S+MBCs (%), median (IQR)	0,04 (0-15)	0 (0-0,07)	0,310	0,20 (0,15-0,28)	0,13 (0,02-0,20)	0,246
S+ATMs (%), median (IQR)	0 (0-0,07)	0,06 (0-0,38)	0,280	0,08 (0,04-0,18)	0 (0-0)	0,065
**COVID-19**						
Days of SARS-CoV-2 qPCR positivity	NAp	NAp	NAp	10 (9-12)	30 (22.5-52)	0,073
Severe/moderate COVID19	NAp	NAp	NAp	2 (40)	7 (29)	0,633
Pneumonia	NAp	NAp	NAp	2 (40)	3 (14)	0,195

Abbreviation CLD chronic lung disease, IQR interquartile range, S+ MBC memory B-cells, S+ ATM Atypical Memory B cells.
